# Temporal-specific complexity of spiking patterns in spontaneous activity induced by a dual complex network structure

**DOI:** 10.1038/s41598-019-49286-8

**Published:** 2019-09-04

**Authors:** Sou Nobukawa, Haruhiko Nishimura, Teruya Yamanishi

**Affiliations:** 1 Department of Computer Science, Chiba Institute of Technology, 2-17-1 Tsudanuma, Narashino, Chiba 275-0016 Japan; 2 Graduate School of Applied Informatics, University of Hyogo, 7-1-28 Chuo-ku, Kobe, Hyogo 650-8588 Japan; 3 AI & IoT Center, Department of Management and Information Sciences, Fukui University of Technology, 3-6-1 Gakuen, Fukui, 910-8505 Japan

**Keywords:** Dynamical systems, Network models

## Abstract

Temporal fluctuation of neural activity in the brain has an important function in optimal information processing. Spontaneous activity is a source of such fluctuation. The distribution of excitatory postsynaptic potentials (EPSPs) between cortical pyramidal neurons can follow a log-normal distribution. Recent studies have shown that networks connected by weak synapses exhibit characteristics of a random network, whereas networks connected by strong synapses have small-world characteristics of small path lengths and large cluster coefficients. To investigate the relationship between temporal complexity spontaneous activity and structural network duality in synaptic connections, we executed a simulation study using the leaky integrate-and-fire spiking neural network with log-normal synaptic weight distribution for the EPSPs and duality of synaptic connectivity, depending on synaptic weight. We conducted multiscale entropy analysis of the temporal spiking activity. Our simulation demonstrated that, when strong synaptic connections approach a small-world network, specific spiking patterns arise during irregular spatio-temporal spiking activity, and the complexity at the large temporal scale (i.e., slow frequency) is enhanced. Moreover, we confirmed through a surrogate data analysis that slow temporal dynamics reflect a deterministic process in the spiking neural networks. This modelling approach may improve the understanding of the spatio-temporal complex neural activity in the brain.

## Introduction

Recent progress in studies using neuroimaging modalities has elucidated the structure of the whole network of the brain, which is composed of individual regions and connections, called a connectome^1,2^. In communications among neuronal populations or among brain regions, feedback loops at multiple hierarchical levels of cortical processing can produce spatio-temporal complex behaviours that correspond to brain activity influenced by recurrent neural processes^3,4^. In a recent modelling approach, which utilises network structures such as topological features and coupling strength estimated by neuroimaging modalities, whole-brain spiking neural networks have been constructed and reproduced complex neural behaviours^5,6^. While, despite the intra-regional cortical networks level without these hierarchical topological structures, the neural network exhibits various types of complex spatio-temporal behaviours^7–15^. To describe these behaviours, randomly connected network models, which are approximations for complex cortical networks, were initially proposed, and produced highly irregular behaviours through mutual interactions^7–10^. Moreover, these models, which include physiological cortical network structures such as electrical coupling, excitatory and inhibitory neural populations, spiking neurons, and symmetricity in random networks, can reproduce various types of spatio-temporal neural activity with higher physiological validations in comparison with random networks^11–15^.

With regard to the typical behaviour of the neural activity in the cortex, this network represents the sustaining electrical dynamic fluctuation, despite the nonexistence of sensory stimulation, which is called spontaneous activity^16,17^. This neural activity has several major characteristics: irregular low-frequency neural spiking (≈1 Hz) with high coherent spike transmission between specific pairs of neurons, and high coherent spiking activity between excitatory/inhibitory neural populations^18–20^. Furthermore, the membrane potential behaviour under the threshold exhibits two particularly different states in spontaneous activity: the ‘upstate’ in which the membrane potential depolarizes near the threshold of spiking, and the ‘downstate’ in which the membrane potential hyperpolarizes and spikes cannot arise^18–20^.

Several studies over the last decade have used spiking neuron models to investigate the genesis of this spontaneous activity. Destexhe^21^ showed that spontaneous activity may be produced by the existence of neurons with a low threshold. By using a different approach, investigators in another study showed that the structures of the neural network might induce spontaneous activity^22–24^. For example, Vogles & Abbott and Guo & Li showed that spontaneous activity is produced by the small-world property of synaptic connections characterized by small path lengths and large cluster coefficients and sparseness of random synaptic connections, respectively^22,23^. Of note, the model—which focuses on the distribution of the strength of cortical excitatory synaptic connections, as developed by Teramae *et al*.^24^ —satisfies the physiological characteristics of spontaneous activity with high relevance.

In practical cerebral cortex, excitatory post-synaptic potentials (EPSPs), which is the increasing membrane potential by the pre-synaptic spikes in the excitatory synaptic connections, between cortical pyramidal neurons in a large majority of synapses exhibit potentials in the sub-millivolt range, whereas a sliver of synapses exhibit huge EPSPs (≳1.0 mV)^25,26^. In refs^25,26^, the distribution of EPSPs might follow a log-normal distribution. Regarding the mechanism that produce the spontaneous activity model proposed by Terame *et al*., spikes from most weak synapses (as noise) enhance spike transmission generated from a small number of strong synapses. An interpretation of this phenomenon could be that the mechanism of stochastic resonance induces spontaneous activity^24^. Furthermore, the EPSPs log-normal distribution may enhance the ability of associative memory recall^27^. It can also induce burst spiking, which has a vital function during hippocampal memory consolidation^28^.

Model-based studies of the spiking neural network with small-world properties have revealed that the complexity of neural activity is enhanced by the small-world network structure. Riecke *et al*.^29^ demonstrated that complex spiking activity is induced by the small-world characteristics of excitable spiking neural networks through the rewiring process illustrated by the Watts and Strogatz model^30^. Shanahan^31^ incorporated the physiological clustering features of the cortex into the spiking neural network by using the modular small-world network method^32^. Moreover, Watanabe *et al*. reported that the cerebral cortex exhibits a dual complex network structure, depending on the amount of EPSPs^33^. Weak synaptic networks and strong synaptic networks exhibit random network characteristics and small-world characteristics, respectively^33^. Under this circumstance, the functionalities for the duality of synaptic connectivity and the fluctuation of spontaneous activity have been of recent focus^34–36^.

In our previous study, the spiking neural network model for spontaneous activity^24^ was expanded, and incorporated the previously described duality of synaptic connectivity, characterized by randomness and by small-worldness^37^. We attempted to demonstrate a tendency of enhancement of the complexity of spiking activity on a slow-temporal scale under the existing duality of synaptic connectivity^37^. However, physiological validation of the reproduced spontaneous activity remains to be achieved. In addition, the mechanism underlying this enhancement of complexity has not been elucidated.

Therefore, in this study, based on the outcome of our preliminary previous study^37^, we investigated the spontaneous activity in spiking neural networks using the duality of complex network structures, regarding these aforementioned points. First, we examined the physiological validity of the reproduced spontaneous activity at each duality level with respect to the low firing rate, coherence in spiking activity between excitatory/inhibitory neural populations, and coherence in the temporal spike series among neurons. Second, we analysed the multi-scale entropy (MSE)^38^ of the time series of spontaneous activity and evaluated whether the obtained MSE reflected the dynamics of the spiking neural network using surrogate data analysis. Third, the mechanism for the enhancement of complexity was evaluated with a focus on the structures of spiking neural networks from the local network level and the entire topological level.

## Methods

### Spiking neural network with dual complex network structure

In this study, based on the spiking neural network with a log-normal EPSPs distribution proposed for producing the spontaneous activity^24^, the spiking neural network with a dual complex network structure, depending on synaptic weight, were constructed. In this network, we used the conductance-based leaky integrate-and-fire neuron model, to describe the membrane potential *v*(*t*):

1 $$\frac{dv}{dt}=-\frac{1}{{\tau }_{m}}(v-{V}_{L})-{g}_{E}(v-{V}_{E})-{g}_{I}(v-{V}_{I})+{I}_{{\rm{ex}}},$$

2 $${\rm{if}}\,v\ge {V}_{{\rm{thr}}}\,{\rm{mV}},\,{\rm{then}}\,v(t)\to {V}_{r},$$

in which *τ*_*m*_ is the decay constant of membrane. Also represented were the reversal potentials of the -amino-3-hydroxy-5-methyl-4-isoxazolepropionic acid (AMPA) receptor-mediated excitatory synaptic current, *V*_*E*_; inhibitory synaptic current, *V*_*I*_; leak current, *V*_*L*_; and after-spike reset value of the *v*(*t*), *V*_*r*_. The *I*_ex_ is the signal for external spikes triggering spontaneous activity, which is followed by *A*_*s*_*δ*(*t* − *t*_ex_) mV. In this case, input time, *t*_ex_, is generated by the Poisson process, with a spiking rate of Λ Hz during [0:100] ms. The *A*_*s*_ was set to *V*_thr_ − *V*_*L*_ + 1 to generate a spike from the resting state. The conductances for the excitatory synapse and inhibitory (represented by *g*_*E*_(*t*) [ms^−1^] and *g*_*I*_(*t*) [ms^−1^], respectively) are given by

3 $$\frac{d{g}_{X}}{dt}=-\frac{{g}_{X}}{{\tau }_{s}}+\sum _{j}\,{G}_{X,j}\sum _{{s}_{j}}\,\delta (t-{s}_{j}-{d}_{j}),\,X=E,I.$$

In this case, *τ*_*s*_ is the synaptic decay constant. The *s*_*j*_ is the spike time of the synaptic input from the *j*-th neuron. This spike has the synaptic delay, *d*_*j*_, and is enhanced by the excitatory synaptic weight, *G*_*E*,*j*_ or diminished by the inhibitory synaptic weight, *G*_*I*,*j*_. In our study, parameter sets were used, as follows: *V*_*I*_ = −80 mV; *V*_*L*_ = −70 mV; *V*_*r*_ = −60 mV; *V*_thr_ = −50 mV; *V*_*E*_ = 0 mV; *τ*_*m*_ = 20 ms (excitatory neuron); *τ*_*m*_ = 10 ms (inhibitory neuron); and *τ*_*s*_ = 2 ms). In the numerical simulation, Eq. (3) was solved by the Euler method with a time-step size of Δ*t* = 0.1 ms. The period of refractoriness was set to 1 ms. The synaptic delays in excitatory-to-excitatory connections were set to uniform random values [1:3] ms, and the synaptic delays for other connections were set to [0:2] ms. The sizes of this spiking neural network for excitatory neural population for and for inhibitory neurons were set to *N*_*E*_ = 10000 and *N*_*I*_ = 2000, respectively.

**Figure 1:**
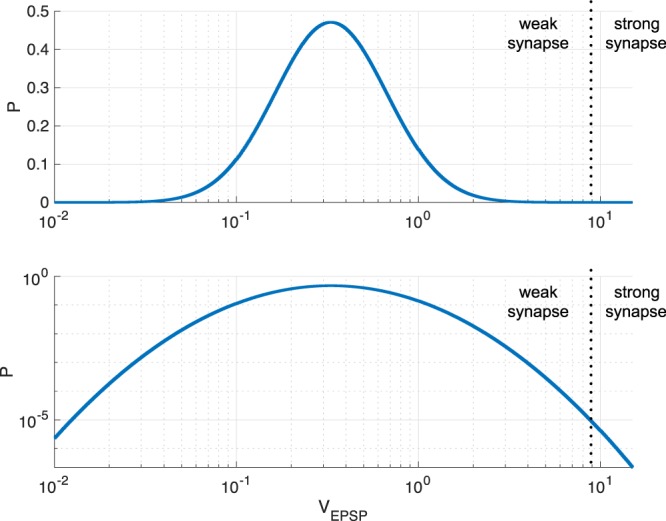
Synaptic weight distribution among excitatory neurons (upper: single logarithmic plot; lower: double logarithmic plot). The dotted line represents the threshold dividing the network structures. EPSP: excitatory postsynaptic potential. (*σ* = 1.0, *μ* − *σ*^2^ = log0.2).

The EPSP amplitudes, *V*_EPSP_ mV, which are increased membrane potentials resulting from the input spikes of excitatory synaptic connections, followed a log-normal distribution (Fig. 1):

4 $$p(x)=\frac{\exp [-\,{(\log x-\mu )}^{2}/2{\sigma }^{2}]}{\sqrt{2\pi }\sigma x}.$$

In this case, *σ* and the mode of the distribution were set to 1.0 and 0.2, respectively (*μ* − *σ*^2^ = log0.2). The values *V*_EPSP_ > 15 mV were declined as unrealistic values, and a new value was produced from the distribution.

Regarding the parameter to determine the topology for the excitatory-to-excitatory network, the number of synaptic connections was set to $0.1{N}_{E}^{2}$. Synaptic connections with *V*_EPSP_ ≤ 9 mV (i.e., weak synapses) were wired by using the Watts-Strogatz model^30^ with a rewiring probability of 1.0, which corresponds a random network. However, synaptic connections with *V*_EPSP_ > 9 mV (i.e., strong synapses) were wired with rewiring probability *β* from the ring type of topology. Each neuron in other synaptic connections was randomly connected; the coupling probability for excitatory connections and for inhibitory connections was 0.1 and 0.5, respectively.

The *V*_EPSP_ is an observable value; therefore, these values must be converted into synaptic weight *G*_*E*_. In this conversion, we considered a simple case in which the spike input from a single excitatory synapse was applied to a post-synaptic neuron at *t* = 0 ms. The behaviour of the membrane potential, *v*(*t*), was as follows:

5 $$\frac{dv(t)}{dt}=-\frac{1}{{\tau }_{m}}(v(t)-{V}_{L})-{g}_{E}(t)(v(t)-{V}_{E}),$$

6 $$\frac{d{g}_{E}(t)}{dt}=-\frac{{g}_{E}(t)}{{\tau }_{s}}+{G}_{E}\delta (t).$$

We numerically solved Eqs (5) and (6). The relationship between *V*_EPSP_ and *G*_*E*_ can be clarified, as follows:

7 $${G}_{E}={V}_{{\rm{EPSP}}}/100.$$

The other synaptic weights were set to 0.018 (for excitatory-to-inhibitory connections), 0.002 (for inhibitory-to-excitatory connections), and 0.0025 (for inhibitory-to-inhibitory connections)^24^. The synaptic transmissions in the excitatory-to-excitatory synaptic connections failed, based on the failing rate, depending on the amplitude of the EPSP: ${P}_{E}=\frac{a}{a+{V}_{{\rm{EPSP}}}}$ (*a* = 0.1 mV)^24,26^. In this study, the spiking neural network was simulated by using Brian2 (https://brian2.readthedocs.io/en/2.0rc/index.html)^39^.

### Evaluation indexes

#### Index to measure neural activity

To measure spiking activity, the spiking rates in the excitatory neural population, *r*_*E*_ Hz, and the inhibitory neural population, *r*_*I*_ Hz, were used:

8 $${r}_{X}(t)={10}^{3}\frac{{S}_{X}(t)}{\Delta t{N}_{X}}\,X=E,I.$$

In this instance, *S*_*E*_ indicates the spike frequency in the bin in which the temporal width is 0.1 ms in excitatory neural populations, and *S*_*I*_ is the spike frequency for inhibitory neural populations. A Gaussian-shaped window with temporal width 10 ms was applied to *r*_*E*_(*t*) and *r*_*I*_(*t*) to be smoothed in the time series. The period that has a high spiking rate *r*_*E*_ > *θ* Hz, termed ‘the activate period’ in this study, was also used to quantify neural activity.

#### Index for the temporal complexity of neural activity

In this study, MSE^38^ was utilised to quantify the complexity with time-scale dependency in the time series (*r*_*E*_). A sample entropy for a stochastic variable {*x*_1_, *x*_2_, … *x*_*N*_} is given by the following equation:

9 $$h(r,m)=-\,\log \,\frac{{C}_{m+1}(r)}{{C}_{m}(r)},$$

in which *C*_*m*_(*r*) represents the probability of satisfying the condition with $|{{\bf{x}}}_{i}^{m}-{{\bf{x}}}_{j}^{m}| < r$ (*i*, *j* = 1, 2, …; *i* ≠ *j*). In this case, ${{\bf{x}}}_{i}^{m}$ is a vector with *m* dimension, given by

10 $${{\bf{x}}}_{i}^{m}=\{{x}_{i},{x}_{i+1},\cdots ,{x}_{i+m}\}.$$

To evaluate temporal scale dependencies, a coarse-grained process for {*x*_1_, *x*_2_, …, *x*_*N*_} with the scale factor *τ* (*τ* = 1, 2, …) was applied in the MSE analysis:

11 $${y}_{j}^{(\tau )}=\frac{1}{\tau }\mathop{\sum }\limits_{i=(j-1)\tau +1}^{{j}_{\tau }}\,{x}_{i}.\,(1\le j\le N/\tau )$$

Against the coarse-grained time series, sample entropy *h*^*τ*^(*r*, *m*) was measured. For example, in the case of *τ* = 2, $\{{y}_{j}^{(2)}\}=\{\frac{{x}_{1}+{x}_{2}}{2},\frac{{x}_{3}+{x}_{4}}{2},\cdots ,\frac{{x}_{N-1}+{x}_{N}}{2}\}$. Owing to the dependency of *h*^*τ*^(*r*, *m*) on the scale factor *τ*, we evaluated the characteristics of the complexity in the time series of the excitatory firing rate *r*_*E*_(*t*) in 500 ≤ *t* ≤ 9500 [ms]. In our approach, we set *m* = 2^38^ and set the width of scale at 1 ms. The value for *r* was set to 1.0 to fix the focusing patterns of *r*_*E*_, regardless of the value range. The MSE analysis was executed against 10 trials.

#### Indexes for topological features in synaptic connections

The topology of strong synaptic network (*V*_EPSP_ > 9 mV), which consists of strong synapses as the edges and excitatory neurons as the nodes, were evaluated with regard to clustering, path length, and edge distribution. In this evaluation, a binarized adjacency matrix {*a*_*ij*_} (*i*, *j* = 1, 2, …, *N*_*E*_) for strong synaptic connections was used.

The average clustering coefficient (*CC*) was defined by

12 $$CC=\frac{1}{{N}_{E}}\sum _{i\in {N}_{E}}\,\frac{2{C}_{i}}{{k}_{i}({k}_{i}-1)},$$

in which *k*_*i*_ and *C*_*i*_ represent the degree of a node *i*, given by ${k}_{i}\sum _{j\in {N}_{E}}\,{a}_{ij}$, and the number of triangles around node *i*, respectively.

The average characteristic path length (*PL*) was defined as the average number of edges in the shortest path between two nodes in the network, as follows:

13 $$PL=\frac{1}{{N}_{E}({N}_{E}-1)}\sum _{i,j\in {N}_{E},j\ne i}\,{d}_{ij},$$

in which the matrix for the shortest path length between nodes *i* and *j* are represented by {*d*_*ij*_}. In the Watts–Strogatz model, the distribution of the degree of edges changes, depending on *β*. Therefore, we assessed the degree of node *i* using *D*_*i*_. To observe the dependency of topological changes on rewiring properties *β* in the Watts–Strogatz model, the normalized average clustering coefficient was used, based on the *β* = 0 case [*CC*(*β*)/*CC*(0) and *PL*(*β*)/*PL*(0)].

#### Surrogate data analysis

We derived surrogate data using the iterative amplitude-adjusted Fourier transformed (IAAFT) surrogate data analysis^40^ for the spiking rate, *r*_*E*_, to examine whether a nonlinear dynamic process was involved in the spiking. The iteration number was set to 50, and 10 IAAFT surrogate datasets were generated by different random seeds per original *r*_*E*_. These values of sample entropy *h*^*τ*^(*r*, *m*) were averaged and compared with the value from the original *r*_*E*_. To compare the sample entropies for the original data and those for IAAFT, we used the paired-sample *t*-test. The number of paired samples was set to 10. Two-tailed *α* levels of 10^−2^ and 10^−3^ were considered statistically significant.

## Results

### Characteristics of spontaneous activity and its physiological validation

**Figure 2:**
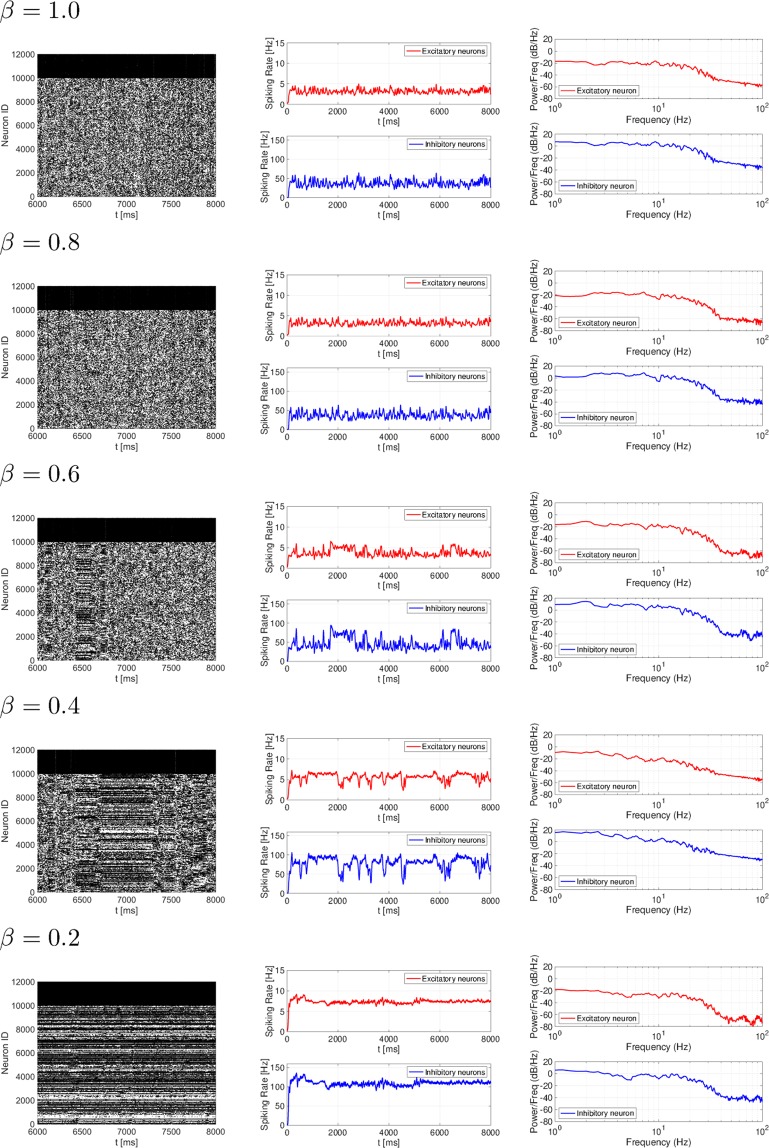
Rewiring probability *β* dependency of spiking activity in a spiking neural network with small-world network for strong synaptic connections and random network for weak synaptic connections (*β* = 1.0, 0.8, 0.6, 0.4, 0.2). The left, middle, and right images represent the raster plot, spiking rate time-series for *r*_*E*_ (upper) and *r*_*I*_ (lower), and their power spectrum, respectively. In the *β* = 1.0, 0.8 case, an irregular spatio-temporal spike pattern was observed. With decreasing *β* values (*β* = 0.6, 0.4, 0.2), constant spike patterns with a higher spiking rate [*r*_*E*_ ≈ 5.0 Hz, (*r*_*I*_ ≈ 70 Hz) in the *β* = 0.6 case, *r*_*E*_ ≈ 6.0 Hz (*r*_*I*_ ≈ 100 Hz) in the *β* = 0.4 case, and *r*_*E*_ ≈ 7.0 Hz (*r*_*I*_ ≈ 110 Hz) in the *β* = 0.2 case] were maintained in a specific duration.

The temporal characteristics of spiking activity was evaluated in the spiking neural network with a coexisting small-world network for strong synaptic connections and random network for weak synaptic connections under the conditions of different rewiring probabilities in strong synaptic connections: *β* = 1.0, 0.8, 0.6, 0.4, 0.2. The raster plot, time series of spiking rates of *r*_*E*_ and *r*_*I*_, and power spectrum for spiking rates are shown in Fig. 2. In the *β* = 1.0, 0.8 case in which the topologies of a strong synaptic network (*V*_EPSP_ > 9 mV) exhibits a nearly random network corresponding to the topologies of a weak synaptic network (*V*_EPSP_ ≤ 9 mV), an irregular spatio-temporal spike pattern was shown in the raster plot. The time series of spiking rates exhibited irregular oscillations around *r*_*E*_ ≈ 4.0 Hz and *r*_*I*_ ≈ 40 Hz. Moreover, constant spike patterns arose in a specific duration in cases of a smaller *β* value (*β* = 0.6, 0.4, 0.2) such as [6500:6600] ms for *β* = 0.6, [6600:7250] ms for *β* = 0.4, and [6000:8000] for *β* = 0.2 (see the raster plot). The spiking rate was increased for *r*_*E*_, *r*_*I*_, compared with cases in which *β* = 1.0, 0.8 (*r*_*E*_ ≈ 5.0 Hz and *r*_*I*_ ≈ 70 Hz for *β* = 0.6; *r*_*E*_ ≈ 6.0 Hz and *r*_*I*_ ≈ 100 Hz for *β* = 0.4; and *r*_*E*_ ≈ 7.0 Hz and *r*_*I*_ ≈ 110 Hz for *β* = 0.2). In addition, the power spectrum of the spiking rates for *β* = 1.0, 0.8, 0.6, 0.4, 0.2 was mostly distributed in the region $\lesssim 40$ Hz (power/Freq ≳ −40 [dB/Hz]).

**Figure 3:**
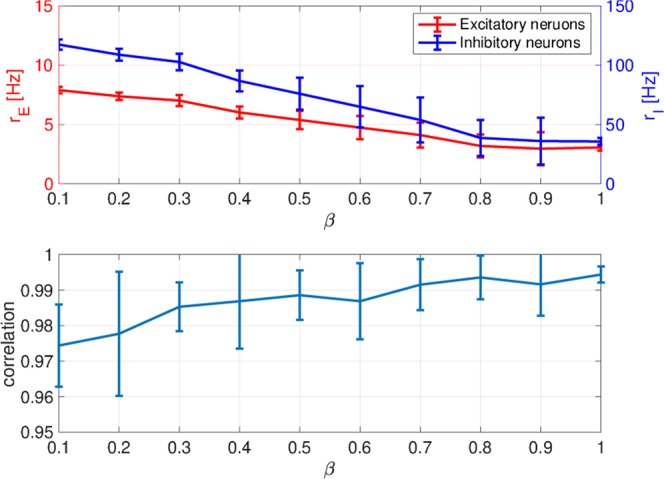
The mean spiking rate in excitatory/inhibitory neural populations (upper) and the correlation between the time series of the excitatory spiking rate *r*_*E*_ and that of the inhibitory spiking rate *r*_*I*_ (lower). For all *β* cases, neurons exhibit low firing rates (${r}_{E}\lesssim 0$ Hz, ${r}_{I}\lesssim 100$ Hz) and high coherent spiking activity between the excitatory and inhibitory neural populations.

**Figure 4:**
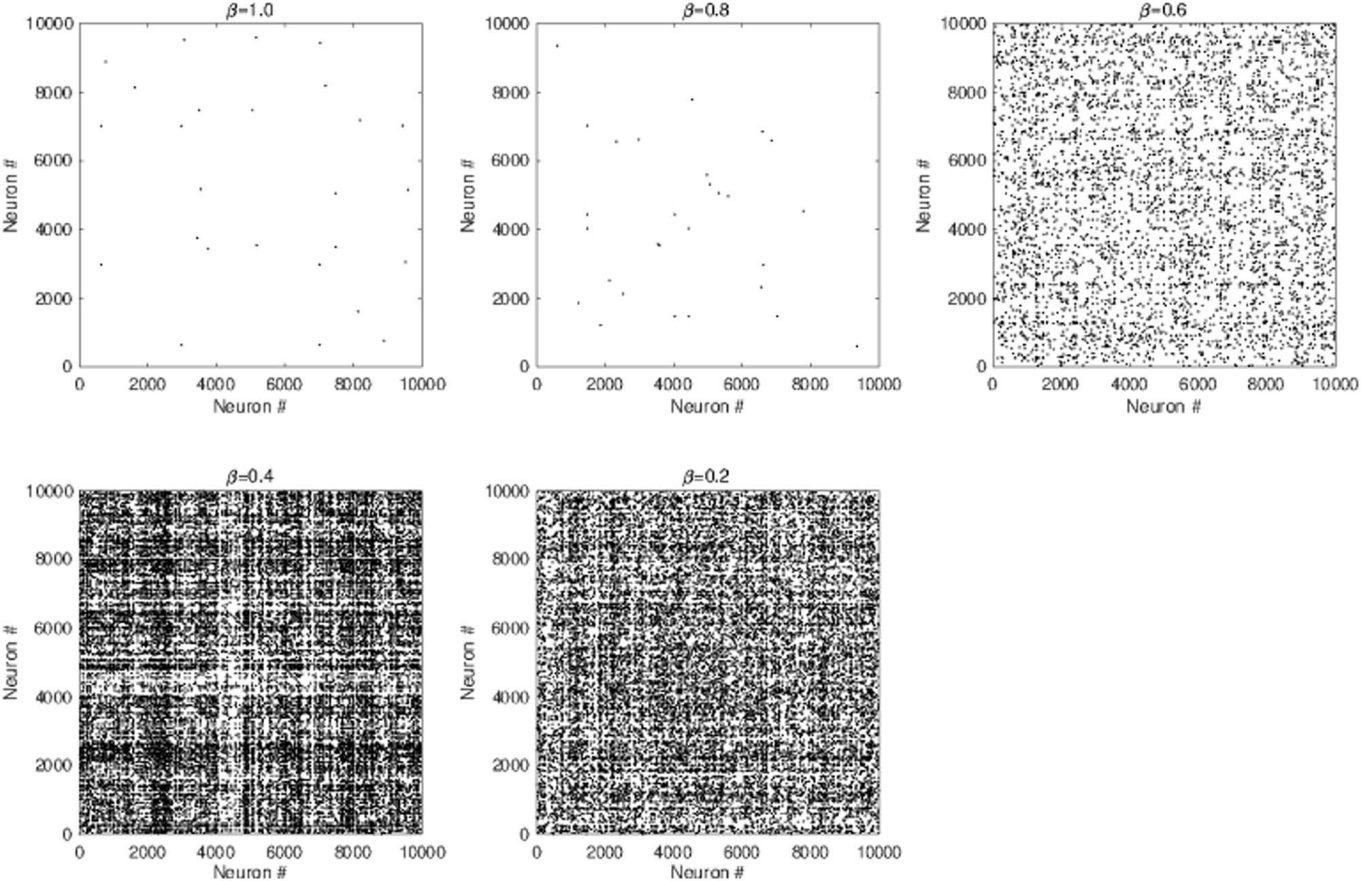
Neuron pairs satisfying the high correlation among the temporal spike series. The black dots indicate neuron pairs with Pearson’s correlation over 0.5. High coherent spike transmission between specific neurons. Rates among pairs with Pearson’s correlation over 0.5 in all pairs connected by strong synapses are 2.6 × 10^−7^ (*β* = 1.0), 2.8 × 10^−7^ (*β* = 0.8), 4.9 × 10^−5^ (*β* = 0.6), 4.2 × 10^−4^ (*β* = 0.4), and 2.4 × 10^−4^ (*β* = 0.2).

To examine whether the spiking activity at each *β* value was consistent with actual physiological spontaneous activity^18–20^, we evaluated the spiking rates, the correlation of temporal excitatory/inhibitory spiking activity, and the correlation among the temporal-spike series. Figures 3 and 4 depict the mean spiking rate, temporal correlation between excitatory/inhibitory neural populations, and the correlation among the temporal-spike series of excitatory neurons. Based on these results, we could observe that in all *β* cases, neurons exhibited low firing rates (${r}_{E}\lesssim 10$ Hz, ${r}_{I}\lesssim 100$ Hz), high coherent spike transmission between specific neurons (the rate between highly coherent pairs with Pearson’s correlation over 0.5. In addition, all pairs connected by the strong synapses were less than 4.2 × 10^−4^), and high coherent spiking activity between excitatory/inhibitory neural populations (Pearson’s correlation was approximately 0.98). This finding was consistent with the physiological findings^18–20^. These physiological conditions were primarily determined by lognormal-distribution of EPSPs^24^, whereas the dual complex network structure virtually did not affect them.

### The MSE analysis for the time series of spontaneous activity

**Figure 5:**
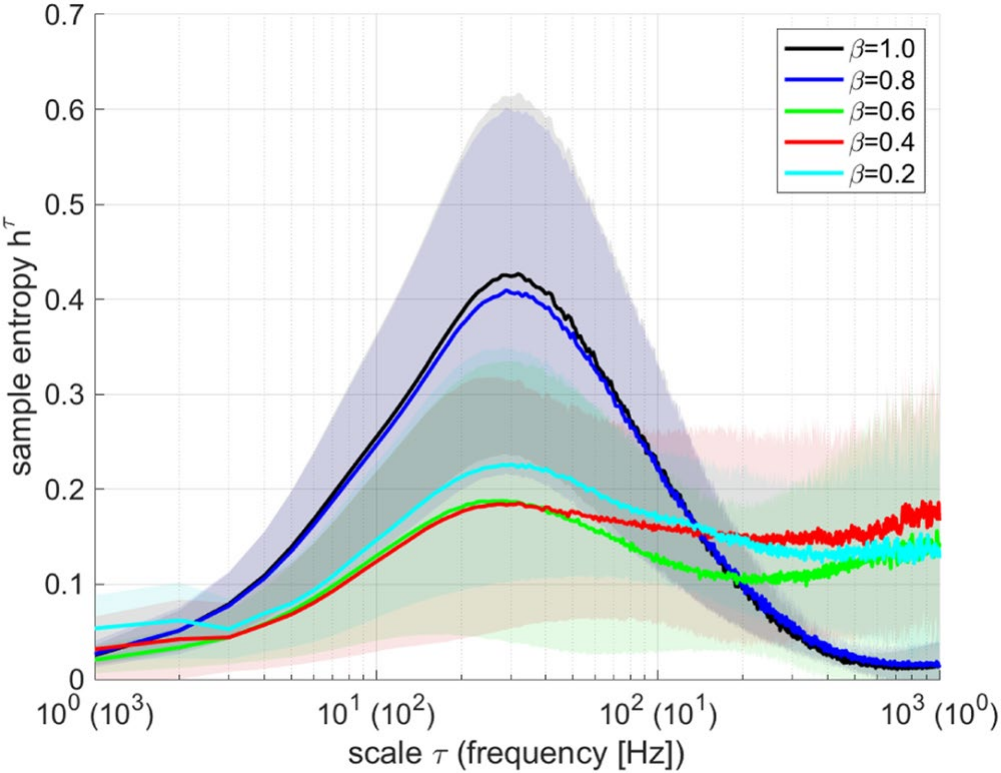
Profile of multiscale entropy as a function of temporal scale *τ* for the time series of the excitatory spiking rate *r*_*E*_ (the width of the temporal scale is 1 ms). The mean and standard deviation between trials are represented by the solid and shaded areas, respectively. In the *β* = 1.0, 0.8 case, *h*^*τ*^ exhibits a peak at *τ* ≈ 20 (50 Hz). With decreasing *β* values such as *β* = 0.6, 0.4, 0.2, the *h*^*τ*^ in the larger *τ* (i.e., small frequency) regions increases (*τ* ≳ 200 (5 Hz)).

The complexity of temporal scale dependency for the time series of the spiking rate *r*_*E*_, corresponding to the results in Fig. 2, was analysed by MSE (given by Eqs 9 and 10). Figure 5 shows the profile of the sample entropy *h*^*τ*^ of *r*_*E*_ as function of temporal scale *τ* (corresponding frequency of 1/(10^−3^*τ*) Hz) for *β* = 1.0, 0.8, 0.6, 0.4, 0.2. In the *β* = 1.0, 0.8 case, dependency of *h*^*τ*^ on temporal scale had a unimodal maximum peak at *τ* ≈ 20 (50 Hz). In smaller *β* cases (e.g., *β* = 0.6, 0.4, 0.2), the *h*^*τ*^ increased at larger *τ* (i.e., small frequency) regions (*τ* ≳ 200 (5 Hz)). This characteristic was the same with the tendency at single trial shown in our previous preliminary results^37^.

**Figure 6:**
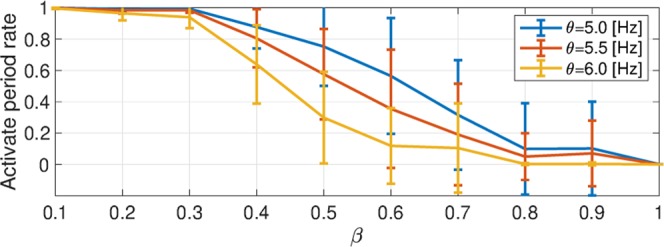
Dependence of the average of the activate period for the excitatory neural population (i.e., satisfies *r*_*E*_ > *θ* Hz) on rewiring probability *β*. The activate period rate decreases with increasing *β* value.

The mechanism for the enhancement of complexity at a larger temporal scale was then evaluated. Figure 6 shows the activate period rate during the evaluation period as a function of *β*. The activate period rate decreased with increasing *β* value. Hence, the enhancement of complexity with the large temporal scale, as presented in Fig. 5, arose at a moderate activate period rate because of the intermittent appearance of the active state (see also Fig. 2).

**Figure 7:**
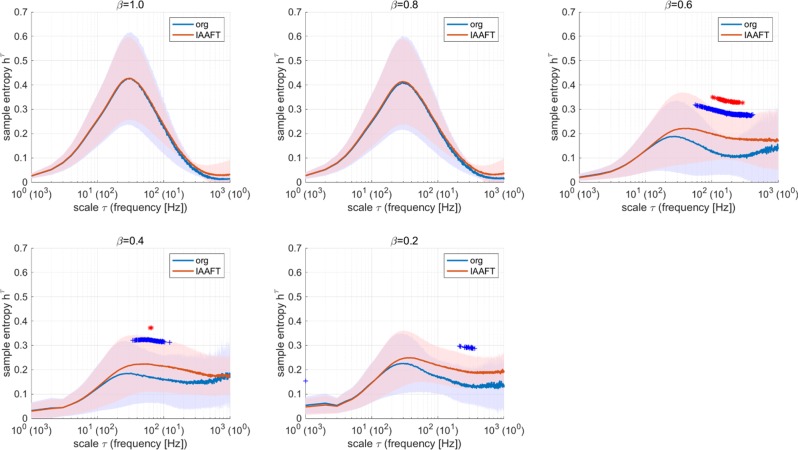
Comparison between multiscale entropy profiles of the original *r*_*E*_ and those of the IAAFT surrogate data. The cases in which differences in *h*^*τ*^ are statically significant are represented by blue (^+^*p* < 10^−2^) and red (*p < 10^−3^). A significant difference between the original/IAAFT ones is observed at *τ* ≈ 200 (5 Hz) in cases of *β* = 0.6, 0.4, 0.2; the MSE profile at large temporal scales reflects a nonlinear dynamic process. Org: original; IAFFT: Iterative amplitude-adjusted Fourier transformed.

Furthermore, we evaluated whether the temporal behaviour in spiking activity reflected a nonlinear dynamic process in the spiking neural networks. In the IAAFT process, the power spectrum was maintained, and the phase spectrum was randomised in the frequency space^40^. In comparison with the MSE profile for IAAFT surrogate data, a significant difference was observed at *τ* ≈ 200 (5 Hz) for cases of *β* = 0.6, 0.4, 0.2; the MSE profile under this condition reflected a nonlinear dynamic process (Fig. 7). This finding implied that the MSE profile at a large temporal scale reflected the characteristic of structure for the dual complex spiking network.

### Relationship between spontaneous activity and network topology

**Figure 8:**
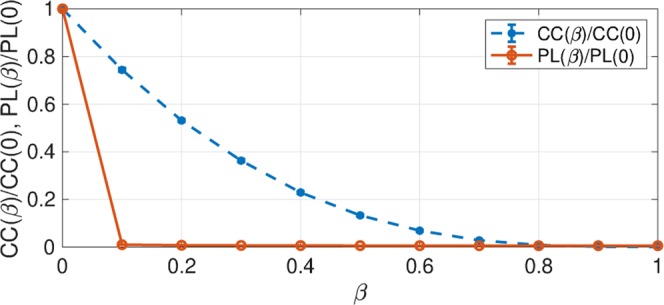
In the strong synaptic network, the normalised clustering coefficient *CC*(*β*)/*CC*(0) and the mean shortest path length *PL*(*β*)/*PL*(0), as a function of the rewiring probability *β*. In the range 0.2 ≤ *β* ≤ 1.0 (which corresponds to the *β* range for changing MSE profiles in Fig. 5), *CC*(*β*)/*CC*(0) decreases from 0.52 to 0, whereas *PL*(*β*)/*PL*(0) remains at approximately 0.

We investigated the relationship between the MSE profile and the topology of a strong synaptic network (*V*_EPSP_ > 9 mV) by clustering coefficient and the mean shortest path length. Figure 8 shows the normalized clustering coefficient *CC*(*β*)/*CC*(0) (given by Eq. (12)) and mean shortest path length *PL*(*β*)/*PL*(0) (given by Eq. (13)), as a function of the rewiring probability *β*. Our results suggested that in the range 0.2 ≤ *β* ≤ 1.0 in which the *β* range for changing MSE profiles in Fig. 5), *CC*(*β*)/*CC*(0) decreased from 0.52 to 0. However, in this range of *β*, *PL*(*β*)/*PL*(0) was nearly zero^37^.

**Figure 9:**
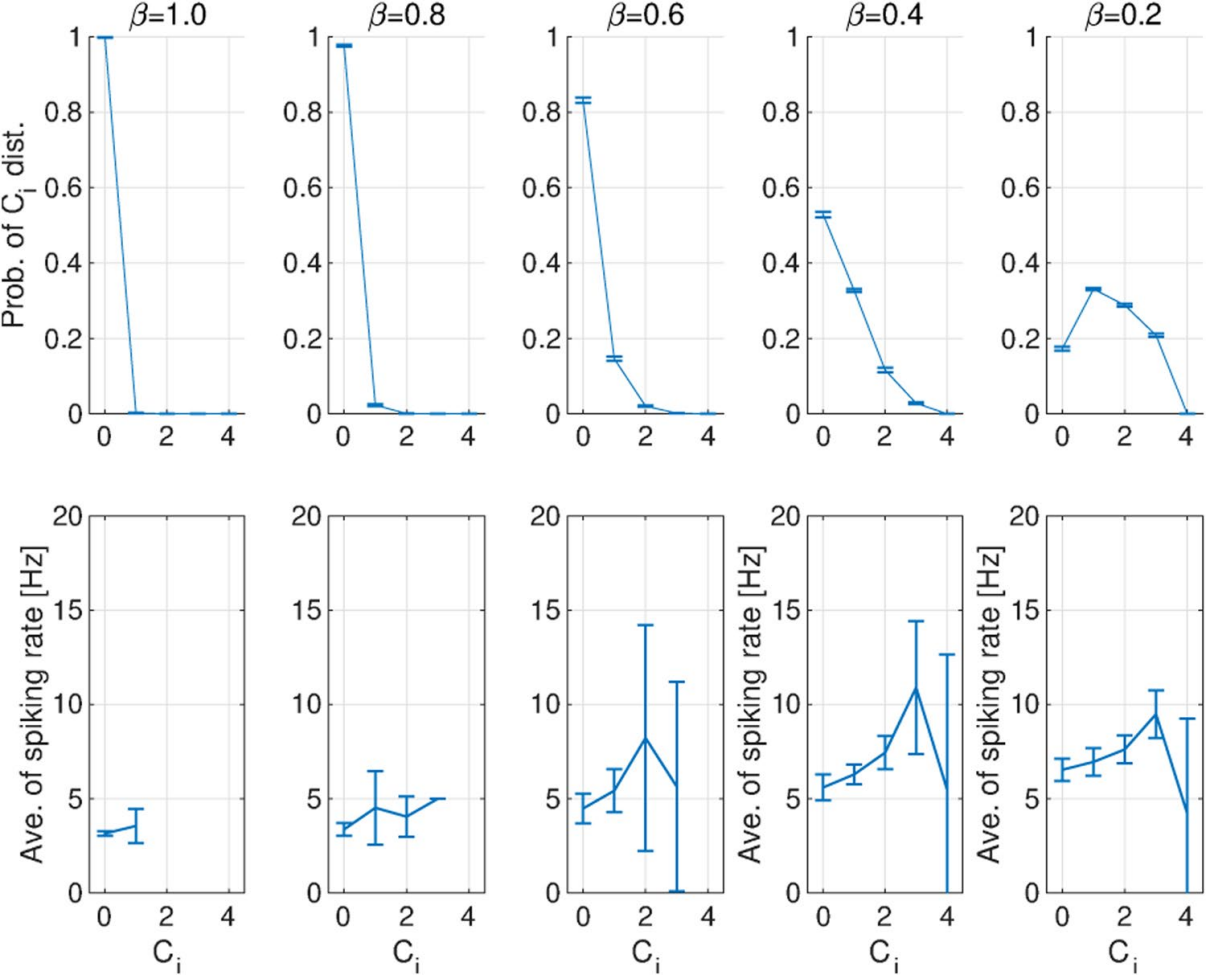
Relationship between spiking activity and clustering structures. Probability distribution of the number of triangles around node *i*: *C*_*i*_ (upper) and dependence of the temporal average spiking rate of the excitatory neural population on *C*_*i*_ (lower). A neuron with high *C*_*i*_ exhibits a tendency toward a high spiking rate, compared with the case of *C*_*i*_ = 0. *C*_*i*_: number of triangles around node *i*. Ave.: average; Prob.: probability; Dist.: distribution.

Our investigation confirmed that a neuron with high *C*_*i*_ exhibits a tendency toward a high spiking rate in comparison with *C*_*i*_ = 0 (see Fig. 9). Park *et al*.^41^ reported that the local clustering properties increased the spiking rate in their small-world spiking neural network. Our results were congruent with these findings. With increasing *β* values, neurons with high *C*_*i*_ decrease, leading to a decrease in the activate period for enhancing *h*^*τ*^ at a large temporal scale.

**Figure 10:**
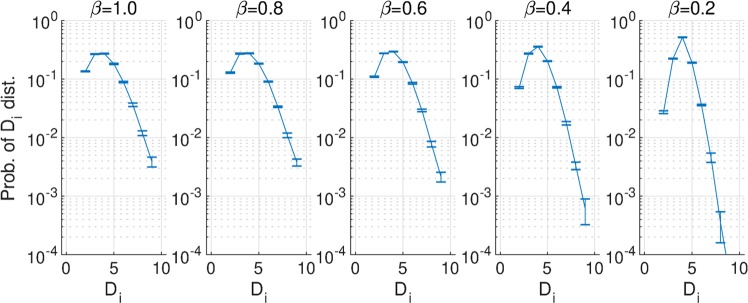
Probability distribution of the number of degrees of node *i*: *D*_*i*_. The number of neurons with high *D*_*i*_ increased with increasing *β* values, but the spiking rate and the activate period decreased. Prob.: probability; Dist.: distribution.

In addition to the local clustering property, we investigated the distributions of the number of degrees *D*_*i*_ around a node *i* and the spiking rate at each *D*_*i*_ (see Fig. 10). The number of neurons with high *D*_*i*_ increased with increasing *β* values, but the spiking rate and the activate period decreased (Figs 3 and 6). Therefore, neurons with a high degree of edge did not contribute to the generation of the activate state or the enhancement of *h*^*τ*^ at a large temporal scale.

## Discussion

In this study, we executed a simulation study using the leaky integrate-and-fire spiking neural network with log-normal synaptic weight distribution in EPSPs and with the duality of complex network consisting of a weak synaptic random network and a strong synaptic small-world network. We found that in cases in which the small-worldness of strong synaptic connections was enhanced, specific spiking patterns arose during temporal irregular spiking activity. The temporal scale profile of MSE for firing rates in the strong synaptic small-world network supported the enhancement of sample entropy at a larger temporal scale (slower frequency) than in cases in which strong synaptic connections approached those of random networks. These results were congruent with the single trial tendency shown in our previous preliminary results^37^. Furthermore, our previous work^37^ mentioned that the clustering coefficient is a possible factor that produces enhancement at a large temporal scale. In this study, by the additional evaluation of local clustering characteristics, we confirmed that this enhancement of the sample entropy at a larger temporal scale was induced by the high spiking frequency of neurons with a high degree of clustering in strong synaptic connections. The IAAFT surrogate data analysis revealed that the sample entropy at the larger temporal scale reflected the dynamics the deterministic behaviour at the larger temporal scale might reflect the structural properties of spiking neural network, typified as the aforementioned strong synaptic clustering characteristic. The spiking activity, more importantly, also satisfied the physiological characteristics of spiking activity such as a low firing rate, high coherence in spiking activity between excitatory/inhibitory neural populations, and high coherence in the temporal-spike series among a few neurons^18–20^. Based on these results, it can be interpreted that the enhancement of complexity at a large temporal scale with the characteristics of the physiological spontaneous activity can be induced by dynamics in the dual complex network structure as a physiological cortical network structure.

With regard to the emergence of various kinds of complex spatio-temporal spiking activity, initial studies^7–10^ of spiking neural networks reported that random connections produced rich dynamics with highly irregular behaviour. Moreover, several studies applied physiological cortical structures to the random spiking network and showed that these expanded random network model can induce various spatio-temporal activities such as the intermittent transition between synchronous and asynchronous states^11–14^. Regarding the phenomena of emerging slow-temporal scale dynamics, Ostojic *et al*., Mastrogiuseppe & Ostojic and Wieland *et al*. reported that in a spiking neural network consisting of excitatory and inhibitory neural populations, irregular fluctuating activity, including slow-temporal scale dynamics, is induced in the strong strength regime of synaptic weight^12,13,15^. Moreover, Mart *et al*. have recently revealed that increasing symmetricity in random networks induces irregular dynamics with the slow-temporal scale^14^. They reported that, in a network with symmetricity (i.e., a portion of neurons have bidirectional connectivity), the neurons easily affect each other, and the neuron’s activity has feedback. These interactions may induce the complex temporal behaviour with slow-temporal dynamics^14^. In our spiking neural network, in the regime of the strong clustering characteristic, the spikes of neurons belonging in same clusters easily affected each other and had feedback. Therefore, a similar effect in the random network with symmetricity^14^ may arise in our spiking neural network.

Future directions and limitations of this study must be considered. During early development in the prenatal period, brain networks undergo enhancement of structural, ordered short-range connectivity. After birth, the short-range connectivity decreases, whereas long-range connectivity appears and strengthens, which leads to a decline in local clustering^42^. Furthermore, an increase in the complexity of the neural activity, as measured by electroencephalogram (EEG)/magnetoencephalography (MEG), is observed during development^43,44^. In particular, Hasegawa *et al*.^44^ revealed that slower temporal complexity increases during early development. The changes that occur after birth can be interpreted as structural and functional integration. However, in Alzheimer’s disease, the degradation of structural connectivity, including the progressive death of neurons, and the development of neurofibrillary tangles and senile plaques, induce a decline in the small-worldness property^45^. This structural degradation induces temporal-specific changes in the complexity of neural activity^4,46–52^. Our model focused on the duality of the cortical neural network structure, and did not consider physiological inter-regional connections. However, by considering region-specific subnetworks and inter-regional connections in this modelling approach may be a useful tool to reveal the relationships between the complexity of microscopic neural activity observed by neuroimaging modalities (e.g. EEG/MEG) and the topology of structural connectivity. Therefore, we plan to develop a spiking neural network consisting of region-specific subnetworks and inter-regional connections to evaluate the relationship between the complexity of neural activity and the topology of structural connectivity.

In conclusion, we conducted a simulation study by using a leaky integrate-and-fire spiking neural network that incorporated the duality of synaptic connectivity of a complex network that follows a log-normal synaptic weight distribution. It has been revealed that this duality has the function of generating complex neural activity, including complex dynamics on a large temporal scale. The future combination of this modelling approach with neuroimaging measures of the brain’s networks and whole-brain network modelling may improve the understanding of the functionality of spatio-temporal complex neural activity.
